# Mitigating continuous cropping barriers in tobacco through organic amendments-induced rhizosphere micro-environmental improvement

**DOI:** 10.3389/fpls.2025.1552955

**Published:** 2025-06-13

**Authors:** Jie Huang, Xinyue Wang, Lili Yang, Yuanhuan Li, Bing Xia, Hailin Li, Xiaohua Deng

**Affiliations:** ^1^ College of Agriculture, Hunan Agricultural University, Changsha, China; ^2^ College of Plant Science and Technology, Hunan Biological and Electromechanical Polytechnic, Changsha, China

**Keywords:** organic fertilizer, rhizosphere, soil amendment, soil microorganisms, environmental stress

## Abstract

Organic amendments supply essential nutrients to crops and act as effective soil conditioners. This study investigates the effects and mechanisms of organic amendments on soil physicochemical properties and microbial diversity, aiming to provide technical solutions for alleviating continuous cropping obstacles. A three-year field experiment was conducted with four types of organic amendments: biological organic fertilizer (BF), carbon-polymerized organic water-soluble fertilizer (CF), potassium fulvate from mineral sources (PF), and microbial fertilizer (MF). The control group received no organic fertilizer application. Results showed that compared to the control, organic amendments significantly increased soil organic matter and available nutrient contents, organic matter and available phosphorus under BF (22.5 and 43.2 mg/kg, respectively) showed increases of 129.6% and 53.7%, respectively. Similarly, available potassium in PF (286.6mg/kg) was elevated by 47.3%. Additionally, soil pH was increased (shifting from 5.4 to 6.0), thereby alleviating soil acidification. 16S rRNA and ITS sequencing revealed that organic amendments markedly influenced soil microbial abundance and diversity, increasing the relative abundance of beneficial bacteria (e.g., The abundance of *Gemmatimonas* rose from 10.0% in control to 19.2% in MF.) while suppressing pathogenic bacteria (e.g., The abundance of *Ralstonia* decreased from 10.5% in control to 2.5% in MF.). In terms of tobacco growth, organic amendments enhanced root length, surface area, volume, and branching number; Significant increases were observed in α-amylase activity (from 0.6 to 1.6 mg/min/g)) and nitrate reductase activity (from 0.15 to 0.21 U/g) in leaves following the treatment.; and reduced the incidence of bacterial wilt and black shank diseases. Specifically, BF, CF, PF, and MF achieved relative control efficacies of 66.7%, 56.0%, 44.0%, and 61.9% against bacterial wilt, and 66.0%, 52.6%, 42.3%, and 55.7% against black shank, respectively. In conclusion, the application of organic amendments can alleviate continuous cropping barriers by improving the soil micro-environment, promoting tobacco growth, and providing certain control over soil-borne diseases. Overall, the BF treatment showed the most comprehensive improvement effect, and to a lesser extent, PF and MF.

## 1 Introduction

Under the backdrop of sustainable agricultural development, enhancing and safeguarding soil quality have emerged as core issues in agricultural production. Soil, serving as the foundation for crop growth, directly influences crop yield and quality through its structure, nutrient content, and biological activity (St-Martin and Bommarco, 2016; Zhang et al., 2022). However, long-term intensive cultivation, excessive use of chemical fertilizers, and irrational land use practices have led to a series of problems such as soil structure degradation, nutrient imbalance, and biodiversity loss (Ali et al., 2020; Awadelkareem et al., 2023), forming continuous cropping barriers that severely constrain the sustainable development of agriculture.

Continuous cropping barriers result from complex factors, including changes in soil physical and chemical properties, variations in the soil ecological environment, and plant autotoxicity. Prolonged continuous cropping and excessive application of physiologically acidic fertilizers can lead to soil acidification (Bai et al., 2019), affecting metal ion concentrations and resulting in restricted crop growth and pathogen proliferation (Li and Liu, 2019; Shen et al., 2024). Continuous cropping also leads to nutrient imbalances, increased concentrations of basic ions in the soil, secondary salinization, and reduced crop yields (Kong et al., 2023). Plant autotoxicity refers to the release of allelochemicals into the soil via root exudates and decomposing residues, which inhibit the growth of the same or related crops (Meng et al., 2023). Additionally, continuous cropping simplifies the soil microbial food web, reduces its stability, and leads to decreased soil biodiversity and disrupted microecological functions (Ali et al., 2020; Yu et al., 2024).

Developing effective strategies to alleviate continuous cropping barriers is crucial for improving agricultural productivity and ensuring sustainable agricultural development. Organic amendments, rich in organic matter, microorganisms, and diverse nutrients, have been widely used in soil improvement and fertility enhancement (Zhang et al., 2021; Leng et al., 2024). The application of organic amendments not only increases soil organic matter content, improves soil structure, and enhances water and nutrient retention but also promotes the reproduction and diversity of soil microorganisms, thereby increasing soil biological activity (Chen et al., 2024; Liu et al., 2024; Nie et al., 2024). Moreover, the organic form of nutrients in organic amendments provides a sustained and stable nutrient supply for crops, reducing the need for chemical fertilizers and minimizing environmental pressure from agricultural activities (Chen et al., 2024; Wang et al., 2024).

Increasing concerns about soil health and agricultural product safety have highlighted the importance of organic amendments in soil amendment. However, existing research has mainly focused on macro-level soil improvement (Ali et al., 2020; Zhang et al., 2021; Leng et al., 2024; Liu et al., 2024; Nie et al., 2024), often with high input costs or short-lived effects (El-Naggar et al., 2019; Lizarralde et al., 2021), while studies on the rhizosphere microenvironment, which has a more direct impact on crop growth, remain limited. The composition of rhizosphere microorganisms is highly complex (Raaijmakers et al., 2009), primarily comprising bacteria, fungi, nematodes, algae, and archaea (Bonkowski et al., 2009). Microbial diversity tends to increase with proximity to the root system (Hartmann et al., 2009). The root system, soil, and microorganisms interact in complex ways. Consequently, modifying the rhizosphere microenvironment can influence crop growth and development (Dessaux et al., 2016), while the application of beneficial microorganisms and organic fertilizers can mitigate continuous cropping obstacles (Zhang et al., 2015), but the specific mechanisms, effects, and interactions with different soil types and crop species require further in-depth investigation. Tobacco, as a vital cash crop, offers significantly higher profitability compared to many traditional crops. Large-scale cultivation can generate employment opportunities and stimulate local economies (Appau et al., 2019). However, tobacco farming depletes soil fertility extensively, leading to soil degradation over prolonged cultivation periods (Gong et al., 2024). Furthermore, tobacco is a crop sensitive to continuous cropping, which promotes the accumulation of soil-borne pathogens and increases susceptibility to diseases (Yu et al., 2024). This study aims to integrate field experiments and laboratory analyses, using dryland tobacco cultivation as a case study. Through preliminary screening, four distinct forms of organic amendments were selected for further field trials, focusing on improving the rhizosphere microenvironment and exploring their impacts on soil nutrient content, acidity indicators, microbial diversity, and crop growth. This research provides a scientific basis for the rational application of organic amendments to alleviate continuous cropping obstacles, promoting soil ecological restoration and sustainable agricultural development.

## 2 Materials and methods

### 2.1 General situation of the experimental area

The experiment was conducted from 2022 to 2024 in Huayuan County, Xiangxi Tujia and Miao Autonomous Prefecture, Hunan Province (28°31′35″N, 109°27′4″E). The study area has an average altitude of 530 meters, with an annual mean temperature of 15.0°C, annual precipitation of 1364 mm, a frost-free period of 279 days, and an annual sunshine duration of 1219 hours. It is characterized by a subtropical monsoon mountainous humid climate. The flue-cured tobacco variety used was Xiangyan 7. The soil type in the experimental area is Ferralsols, with a bulk density of 1.2 g/cm³ and porosity of 53.6%. The soil pH is 5.1. Soil chemical properties include organic matter content of 10.4 g/kg, alkali-hydrolyzable nitrogen of 75.7 mg/kg, available phosphorus of 36.7 mg/kg, and available potassium of 123.1 mg/kg. Additionally, the soil contains hydrolytic acid of 4.1 cmol/kg, exchangeable acid of 3.1 cmol/kg, exchangeable hydrogen of 1.5 cmol/kg, and exchangeable aluminum of 2.6 cmol/kg. The total exchangeable bases and cation exchange capacity are 3.5 cmol/kg and 7.1 cmol/kg, respectively.

### 2.2 Experimental materials

The experimental materials comprised four types of organic substances:

1. Bio-organic fertilizer, organic matter ≥ 47%, containing total nutrients ≥ 8%, and effective viable bacteria count ≥ 0.5 billion/g;
2. Carbon-polymerized organic water-soluble fertilizer, organic matter ≥ 450g/L, protein ≥ 50g/L, and carbon-polymerized polyglutamic acid ≥ 35g/L;
3. Potassium fulvate from mineral sources, organic matter ≥ 50%, containing total nutrients ≥ 10%, humic acid (HA) ≥ 55%, fulvic acid (FA) ≥ 50%, small molecule organic carbon ≥ 45%;
4. Microbial fertilizer, including Bacillus subtilis and Bacillus amyloliquefaciens, with an effective viable bacteria count ≥ 600 billion/g.
5. Other fertilizers: Base fertilizer (N: P_2_O_5_: K_2_O=8: 14: 8), compound fertilizer (N: P_2_O_5_: K_2_O=10: 5: 29), potassium sulfate (K_2_O≥52%).

### 2.3 Experimental design

Four organic fertilizer treatments were established: bio-organic fertilizer (BF), carbon-polymerized organic water-soluble fertilizer (CF), potassium fulvate from mineral sources (PF), and microbial fertilizer (MF). The non-application of organic fertilizer served as the control group. Each treatment was replicated three times. Tobacco plants were arranged with a plant spacing of 0.6 m and a row spacing of 1.2 m, resulting in a plot area of 9.6 m by 10 m (144 m²) in a randomized block design.

The application rates were as follows: 450 kg/ha for bio-organic fertilizer, 75 kg/ha for carbon-polymerized organic water-soluble fertilizer, 15 kg/ha for potassium fulvate from mineral sources, and 2 kg/ha for microbial fertilizer. Bio-organic fertilizer and microbial fertilizer were each uniformly mixed with 750 kg/ha of the special base fertilizer and applied in strips 10 to 15 days before tobacco transplantation (late March). Carbon-polymerized organic water-soluble fertilizer and potassium fulvate from mineral sources were each diluted 500 times with water and applied via irrigation 7 to 10 days after tobacco transplantation (mid-April). Compound fertilizer was applied three times at 150 kg/ha per application between 10 and 30 days after transplanting. Potassium sulfate was applied at 150 kg/ha at 30 days after transplanting, followed by an additional application of 225 kg/ha at 45 days after transplanting. The nitrogen application rate for tobacco was 109.5 kg/ha, with a nutrient ratio of N:P_2_O_5_: K_2_O = 1:1.27:2.73. The experiment was conducted continuously over three years, with samples collected for analysis in 2024.

### 2.4 Measurement indicators and methods

#### 2.4.1 Sampling and detection of rhizosphere soil samples

The five-point sampling method was employed. Sixty days after tobacco transplantation, the rhizosphere soil from five tobacco plants in each plot was collected and thoroughly mixed to form a composite sample. All soil samples were divided into two portions: one portion was stored at -80°C for microbial analysis, and the other portion was air-dried and stored at room temperature for the determination of soil physical and chemical properties.

The total DNA of soil microorganisms was extracted using the UltraClean Microbial DNA Isolation Kit (Mo Bio, United States). PCR amplification was performed on the V3-V4 hypervariable region of bacterial 16S rDNA and the ITS1 region of fungal DNA. Specifically, the forward primer for bacteria was 5′-ACTCCTACGGGAGGCAGCA-3′, and the reverse primer was 5′-GGACTACHVGGGTWTCTAAT-3′; for fungi, the forward primer was 5′-CTTGGTCATTTAGAGGAAGTAA-3′, and the reverse primer was 5′-GCTGCGTTCTTCATCGATGC-3′. Each 25 μL reaction contained 12.5 μL 2× Premix Taq (TaKaRa), 1 μL of each primer (10 μM), 1 μL template DNA, and 9.5 μL ddH_2_O. Thermal cycling conditions were: 95°C for 5 min; 35 cycles of 95°C (30 s), 55°C (30 s), 72°C (45 s); final extension at 72°C for 7 min. Hold at 4°C. The PCR products were evaluated using 1% agarose gel electrophoresis and purified with a DNA purification kit. Qualified PCR products were used to construct sequencing libraries, which were then sequenced on the Illumina HiSeq platform.

The determination of soil physical and chemical properties followed the methods described in reference (Bao, 2023). Soil organic matter was quantified using the potassium dichromate volumetric method; total nitrogen was measured with a Kjeldahl nitrogen analyzer; alkali-hydrolyzable nitrogen was determined by the alkali-hydrolysis diffusion method; available phosphorus was assessed using sodium bicarbonate extraction followed by molybdenum antimony anti-colorimetry; available potassium was determined by ammonium acetate extraction and flame photometry. Soil pH was measured potentiometrically at a soil-to-water ratio of 5:1. Hydrolyzable acidity was determined by sodium acetate extraction and titration with sodium hydroxide. Exchangeable acidity, exchangeable hydrogen, and exchangeable aluminum were determined by potassium chloride exchange and neutralization titration. Cation exchange capacity and total exchangeable bases were determined using the ammonium acetate method.

#### 2.4.2 Collection and analysis of tobacco root systems

Sixty days post-transplantation, three root systems were randomly collected from each plot. The adhering soil was carefully removed from the roots using deionized water, and the root systems were subsequently scanned and analyzed using the WinRHIZO Reg system (WinRHIZO Pro 2022, Regent Instruments Inc., Canada).

#### 2.4.3 Detection of enzyme activities in tobacco leaves

Sixty days post-transplantation, three tobacco plants were randomly selected from each plot. The sixth fully expanded leaf from the top was harvested and immediately preserved in dry ice for transportation to the laboratory. Enzyme activities of nitrate reductase (NR), glutamine synthetase (GS), α-amylase (α-AL), and invertase (Inv) were measured using commercial assay kits provided by Suzhou Comin Biotechnology Co., Ltd.

#### 2.4.4 Investigation on tobacco diseases

The disease investigation was conducted in accordance with reference (Ding, 2018) to determine the incidence rate and disease index of each treatment, and to calculate the disease control efficacy. The specific formulas used are as follows:


$$\begin{array}{c} \text{Morbidity rate }\left(\%\right)\\ =\left(\text{Number of diseased plants}/\text{Total number of investigated plants}\right)\\ \times 100\% \end{array}$$

$$\begin{array}{c} \text{Disease index}=\left(\sum \left(\text{Disease grade}\times \text{ Number of plants at that grade}\right)\right)\\ /(\text{Highest disease grade }\times \text{ Total number of investigated}\\ \text{ plants})\times 100 \end{array}$$

$$\begin{array}{c} \mathrm{Relative field efficiency}\left(\%\right)=((\mathrm{Disease index of the control group}-\\ \text{ Disease index of the treatment group})/\mathrm{Disease index of the control group})\\ \times 100\% \end{array}$$

$$\text{Relative increse }\left(\%\right)=\left(\left({\text{X}}_{\text{treatment}}-{\text{X}}_{\text{control}}\right)/{\text{X}}_{\text{control}}\right)\times 100\%$$

### 2.5 Calculations and statistical analysis

Data processing and statistical analysis were conducted using Excel 2019 and SPSS 25.0. One-way analysis of variance (ANOVA) was used, and multiple comparisons between treatments were performed using Duncan’s method (p < 0.05). The α diversity was analyzed through Shannon index, Chao index, ACE index and Simpson index; the β diversity of microbial communities was evaluated by NMDS (Non-metric Multidimensional Scaling). Stacked bar plots were employed to visualize the taxonomic composition analysis, illustrating genus-level variations in species abundance across samples. Taxa with an average abundance exceeding 1% in all samples were displayed individually for detailed analysis. Remaining taxa were aggregated into an “Others” category, and unannotated entries at the specified taxonomic rank were classified as “Unclassified.” Structural equation modeling (SEM) was conducted using SPSS-AMOS software to investigate the hypothesized pathways through which organic fertilizer influences disease occurrence. Model fit was evaluated using the χ² test, goodness-of-fit index (GFI), and root mean square error of approximation (RMSEA).

## 3 Results

### 3.1 Effects of different organic amendments on soil nutrient composition

The soil nutrient indicators in the tobacco rhizosphere are summarized in **Table 1**. After three years of continuous organic fertilizer application, the soil organic matter content was significantly increased, with the BF (biological organic fertilizer) treatment showing the most substantial improvement, increasing by 129.6% compared to the control (9.8g/kg). The total nitrogen content in the BF and CF (carbon-polymerized organic water-soluble fertilizer) treatments was significantly the highest than that in control. While there were no significant differences in available nitrogen content among treatments, significant variations were observed in available phosphorus and available potassium contents. Specifically, the available phosphorus content in BF, CF, PF (potassium fulvate from mineral sources), and MF (microbial fertilizer) treatments increased by 53.7%, 26.0%, 14.2%, and 37.7%, respectively, compared to control (28.1mg/kg). Similarly, the available potassium content in BF, CF, PF, and MF treatments increased by 29.3%, 14.8%, 47.3%, and 24.3%, respectively, compared to control (194.6mg/kg), among them, PF was significantly higher than other treatments.

**Table 1:** Determination of soil nutrient indicators.

Treatment	OM (g/kg)	TN (g/kg)	AN (mg/kg)	AP (mg/kg)	AK (mg/kg)
Control	9.8 ± 0.9c	7.8 ± 0.7b	103.2 ± 8.2a	28.1 ± 2.2c	194.6 ± 16.9c
BF	22.5 ± 1.2a	10.3 ± 0.8a	111.3 ± 2.8a	43.2 ± 1.2a	251.7 ± 12.4b
CF	20.8 ± 0.7a	9.7 ± 1.4a	117.5 ± 6.4a	35.4 ± 1.8b	223.4 ± 10.5bc
PF	15.7 ± 2.1b	8.4 ± 0.7ab	104.1 ± 3.7a	32.1 ± 2.4bc	286.6 ± 8.8a
MF	14.4 ± 1.7b	8.2 ± 2.1ab	108.4 ± 9.7a	38.7 ± 2.1ab	241.8 ± 22.8b

BF, biological organic fertilizer; CF, carbon-polymerized organic water-soluble fertilizer; PF, potassium fulvate from mineral sources; MF, microbial fertilizer; OM, Organic matter; TN, Total nitrogen; AN, Alkaline hydrolysis nitrogen; AP, Available phosphorus; AK, Available potassium. The values in the table are expressed as (mean ± standard deviation). Different lowercase letters (a, b, c) indicate significant differences among the treatments (P < 0.05).

### 3.2 Effects of different organic amendments on soil acidity indicators

The acidity indicators of tobacco rhizosphere soil are summarized in **Table 2**. The application of BF, CF, and PF significantly increased soil pH and the total exchangeable base content. Specifically, hydrolyzable acidity was significantly reduced in BF, CF, and MF treatments by 50.0%, 42.1%, and 28.9%, respectively. Exchangeable acidity, exchangeable hydrogen, and exchangeable aluminum were also significantly reduced in the organic fertilizer treatments, with BF treatment showing the most pronounced reductions of 34.5%, 63.6%, and 44.4%, respectively. Additionally, the cation exchange capacity (CEC) was significantly enhanced in all organic fertilizer treatments, increasing by 38.0%, 15.2%, 20.3%, and 17.7% for BF, CF, PF, and MF, respectively.

**Table 2:** Determination of soil acidity indicators.

Treatment	pH	HA (cmol/kg)	EA (cmol/kg)	EH^+^ (cmol/kg)	EAl^3+^ (cmol/kg)	EB (cmol/kg)	CEC (cmol/kg)
Control	5.4 ± 0.3c	3.8 ± 0.2a	2.9 ± 0.1a	1.1 ± 0.1a	2.7 ± 0.2a	4.9 ± 0.1b	7.9 ± 0.5b
BF	6.0 ± 0.1a	1.9 ± 0.1c	1.4 ± 0.1c	0.4 ± 0.0b	1.5 ± 0.0b	6.3 ± 0.1a	10.9 ± 0.1a
CF	5.8 ± 0.1ab	2.2 ± 0.0bc	2.1 ± 0.0bc	0.5 ± 0.3b	1.7 ± 0.0b	6.3 ± 0.0a	9.1 ± 0.2a
PF	5.6 ± 0.1b	2.4 ± 0.1b	2.4 ± 0.1b	0.5 ± 0.0b	1.9 ± 0.3b	6.6 ± 0.3b	9.5 ± 0.2a
MF	5.5 ± 0.2bc	2.6 ± 0.0b	2.4 ± 0.0b	0.6 ± 0.1b	2.0 ± 0.0b	4.3 ± 0.0a	9.3 ± 0.2a

BF, biological organic fertilizer; CF, carbon-polymerized organic water-soluble fertilizer; PF, potassium fulvate from mineral sources; MF, microbial fertilizer; HA, Hydrolytic acidity; EA, Exchangeable acid; EH^+^, Exchangeable hydrogen; EAl3+, Exchangeable Al^3+^; EB, Total exchangeable base cations; CEC, Cation exchange capacity. The values in the table are expressed as (mean ± standard deviation). Different lowercase letters (a, b, c) indicate significant differences among the treatments (P < 0.05).

### 3.3 Analysis of soil microbial diversity

The alpha diversity index reflects the abundance and diversity of species within a single sample. The results of the α diversity analysis of the soil microbial community are summarized in **Table 3**. For the bacterial community, the Shannon index was significantly higher in the organic fertilizer treatments (BF and MF), while there were no significant differences in the Simpson index among treatments. Both the Chao1 and Ace indices were significantly higher in the organic fertilizer treatments compared to the control. For the fungal community, the Shannon index was significantly higher in BF and MF treatments compared to control. The Chao1 index was also significantly higher in BF, PF, and MF treatments relative to control. The Simpson index in the MF treatment was significantly lower than that in control, while there were no significant differences in the Ace index among treatments.

**Table 3:** Analysis of the α diversity of bacteria and fungi.

Classification	Treatment	Shannon	Chao1	Ace	Simpson
Bacteria	Control	8.1 ± 0.9b	5758.8 ± 235.4b	5668.5 ± 815.1b	0.7 ± 0.1a
	BF	9.5 ± 0.4a	9647.7 ± 227.7a	9269.7 ± 217.4a	0.7 ± 0.1a
	CF	9.3 ± 0.4ab	9033.8 ± 327.4a	8936.9 ± 331.5a	0.6 ± 0.1a
	PF	9.2 ± 0.7ab	9245.4 ± 97.9a	9192.4 ± 103.1a	0.7 ± 0.2a
	MF	9.8 ± 0.3a	9543.6 ± 149.3a	9490.1 ± 137.3a	0.6 ± 0.1a
Fungi	Control	5.7 ± 0.2b	881.5 ± 80.1b	881.1 ± 37.3a	0.8 ± 0.2a
	BF	6.4 ± 0.3a	936.1 ± 61.7a	934.2 ± 43.6a	0.6 ± 0.1ab
	CF	6.2 ± 0.5ab	895.2 ± 59.1ab	798.1 ± 122a	0.6 ± 0.2ab
	PF	6.1 ± 0.2ab	925.2 ± 33.7a	951.2 ± 33.7a	0.7 ± 0.1ab
	MF	6.5 ± 0.3a	981.2 ± 61.8a	875.4 ± 65.4a	0.5 ± 0.1b

BF, biological organic fertilizer; CF, carbon-polymerized organic water-soluble fertilizer; PF, potassium fulvate from mineral sources; MF, microbial fertilizer. The values in the table are expressed as (mean ± standard deviation). Different lowercase letters (a, b, c) indicate significant differences among the treatments (P < 0.05).

### 3.4 Differential analysis of soil microorganisms

**Figure 1:**
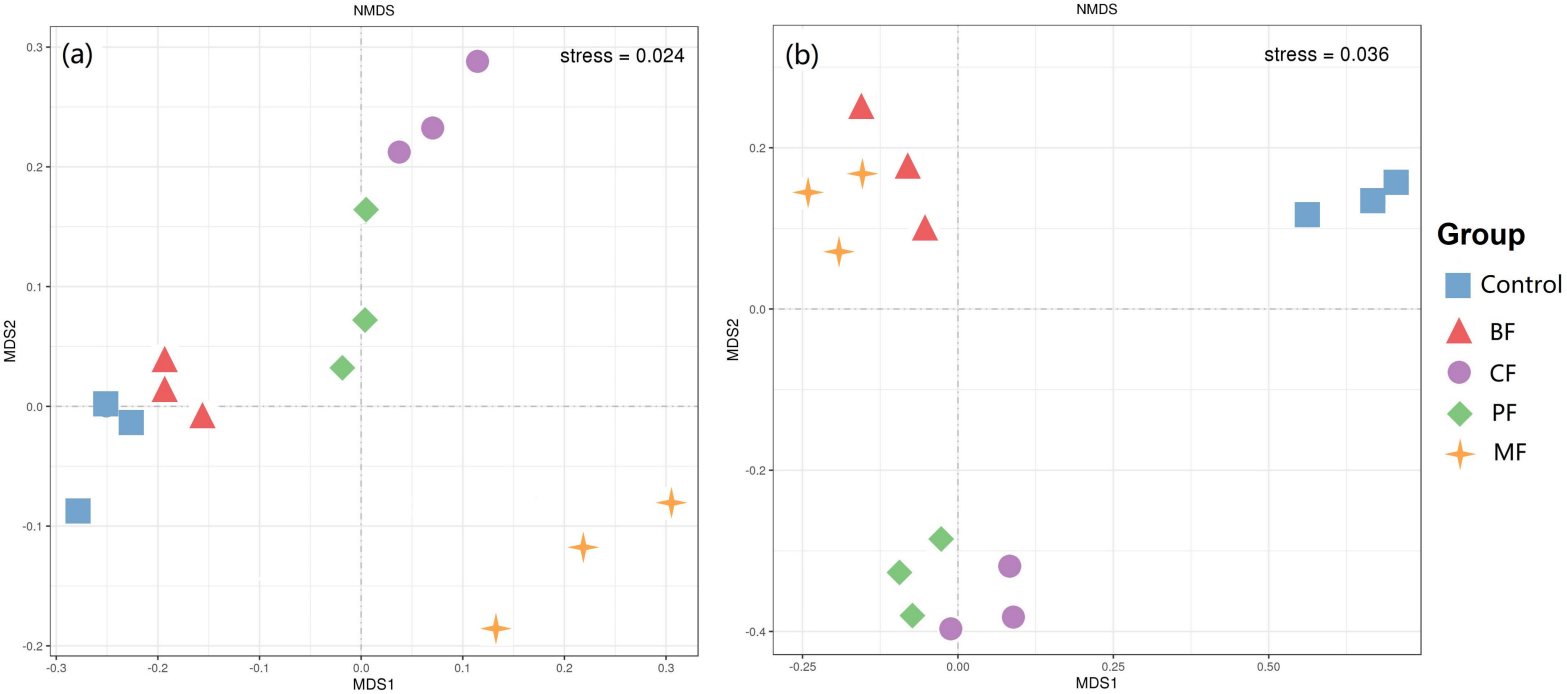
NMDS analysis of bacteria **(a)** and fungi **(b)** in the soil. BF-biological organic fertilizer, CF-carbon-polymerized organic water-soluble fertilizer, PF-potassium fulvate from mineral sources, MF-microbial fertilizer.

The results of the NMDS analysis of the soil microbial community at the genus level are shown in **Figure 1**. The scatter plots of soil bacterial (stress = 0.024) and fungal communities (stress = 0.036) within the same treatment groups exhibit clustered distributions, indicating clear separation between different treatments. This suggests that the application of organic amendments significantly alters the structure of both soil bacterial and fungal communities.

**Figure 2:**
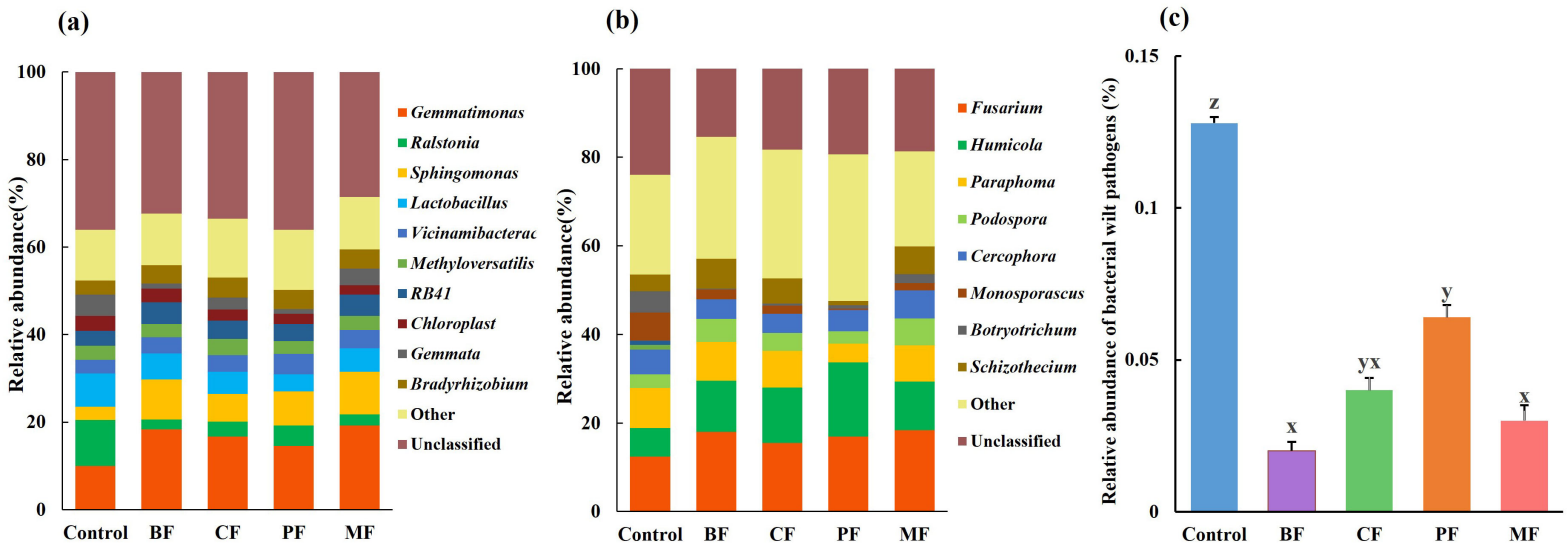
Relative abundance of bacteria **(a)** and fungi **(b)**, Relative abundance of Ralstonia **(c)**. **(c)** BF, biological organic fertilizer; CF, carbon-polymerized organic water-soluble fertilizer; PF, potassium fulvate from mineral sources; MF, microbial fertilizer. Different lowercase letters (z, y, x) indicate significant differences among the treatments (P < 0.05).

Further analysis of the composition of soil bacterial and fungal communities at the genus level is presented in **Figure 2**. The top 10 most abundant genera across all samples are detailed. All other genera are collectively categorized as “Others,” while taxa that could not be classified to a specific level are labeled as “Unclassified.” For bacteria (**Figure 2a**), the dominant genera in control soil include *Ralstonia*, *Gemmatimonas*, Lactobacillus, and *Gemmata*. Compared with control, the relative abundance of *Gemmatimonas* increased significantly in organic fertilizer treatments, specifically: MF (19.2%) > BF (18.3%) > CF (16.7%) > PF (14.5%) > Control (10.0%). Additionally, as shown in **Figure 2c**, the relative abundance of the bacterial wilt pathogen *Ralstonia* decreased significantly after the application of organic amendments.

For fungi (**Figure 2b**), the dominant genera in control soil are *Fusarium, Paraphoma*, and *Monosporascus*. Compared with control, the relative abundance of *Paraphoma* and *Monosporascus* decreased in BF, CF, PF, and MF treatments, while *Fusarium* increased.

### 3.5 Analysis of tobacco root system indicators

**Figure 3:**
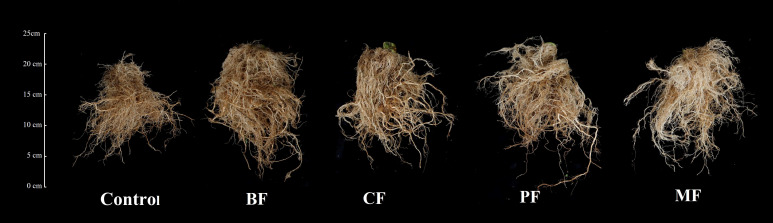
Comparison of tobacco root systems treated with different organic amendments. BF, biological organic fertilizer; CF, carbon-polymerized organic water-soluble fertilizer; PF, potassium fulvate from mineral sources; MF, microbial fertilizer.

**Figure 3** shows the tobacco root systems 60 days after transplantation. Visual inspection indicates that the root systems treated with organic amendments are more robust and dense compared to the control. Quantitative analysis (**Table 4**) reveals that the root length, surface area, and volume of the organic fertilizer treatments are significantly greater than those of control. Specifically, root lengths increased by 29.5% (BF), 15.9% (CF), 25.9% (PF), and 32.4% (MF); surface areas increased by 11.3% (BF), 9.0% (CF), 20.0% (PF), and 23.0% (MF); and volumes increased by 37.1% (BF), 13.1 (CF)%, 25.2% (PF), and 28.8% (MF) in the respective treatments. Among the organic fertilizer treatments, BF, MF, and PF exhibited significantly the longest roots (8263.5, 8040.1 and 8448.4 cm, respectively) compared to CF (7401.2 cm). PF and MF had significantly the largest root surface areas (3623.7 and 3811.9 cm^2^, respectively) than BF (3068.2 cm^2^) and CF (2918.7 cm^2^); BF and MF showed significantly the greatest root volumes (134.2 and 126.1 cm^3^, respectively) than CF (110.7 cm^3^). Additionally, the number of root forks was significantly the highest (129868.3, 180558.7, and 189939.7, respectively) in BF, PF, and MF compared to control (129868.3). No significant differences were observed in the average root diameter.

**Table 4:** Effects of different organic amendments on tobacco root system.

Treatment	Length (cm)	Surface area (cm^2^)	Average diameter (cm)	Volume (cm^3^)	Forks
Control	6382.7 ± 71.5c	2344.2 ± 26.3c	1.4 ± 0.1a	97.9 ± 2.8c	129868.3 ± 454.5b
BF	8263.5 ± 35.4a	3068.2 ± 13.1b	1.2 ± 0.2a	134.2 ± 2.4a	166781.9 ± 713.8a
CF	7401.2 ± 51.1b	2918.7 ± 20.1b	1.1 ± 0.1a	110.7 ± 2.3b	139373.3 ± 961.7b
PF	8040.1 ± 74.4a	3623.7 ± 33.6a	1.4 ± 0.1a	122.6 ± 4.2ab	180558.7 ± 671.8a
MF	8448.4 ± 30.2a	3811.9 ± 13.6a	1.3 ± 0.1a	126.1 ± 1.7a	189939.7 ± 678.4a

BF, biological organic fertilizer; CF, carbon-polymerized organic water-soluble fertilizer; PF, potassium fulvate from mineral sources; MF, microbial fertilizer. The values in the table are expressed as (mean ± standard deviation). Different lowercase letters (a, b, c) indicate significant differences among the treatments (P < 0.05).

### 3.6 Enzyme activity analysis of tobacco leaves

**Figure 4:**
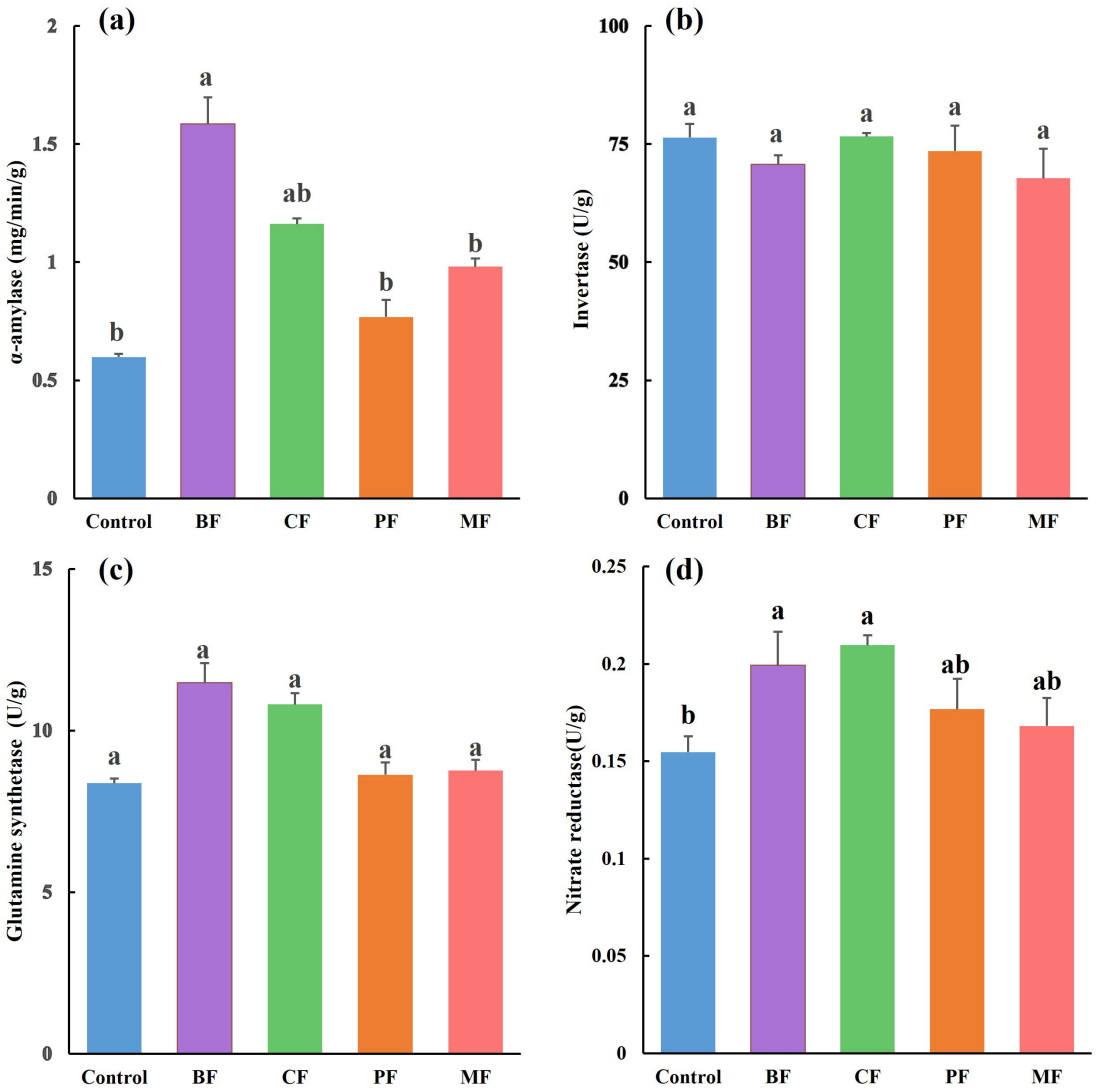
Effect of different organic amendments on enzyme activity in tobacco leaves. **(a)**-amylase, **(b)**- sucrose invertase, **(c)**- glutamine synthetase, (d)-nitratereductase. BF, biological organic fertilizer; CF, carbon-polymerized organic water-soluble fertilizer; PF, potassium fulvate from mineral sources; MF, microbial fertilizer. Different lowercase letters **(a–c)** following the data in the same column indicate significant differences at the 0.05 level (Duncan’s multiple range test).

The results of enzyme activities related to carbon and nitrogen metabolism in tobacco leaves are presented in **Figure 4**. For carbon metabolism-related enzymes, the activity of α-amylase (**Figure 4a**) in the BF (1.59 mg/min/g) was significantly the highest than that in control, PF, and MF, increasing by 165.0%, 106.6% and 61.7%, respectively. The activities of sucrose invertase (**Figure 4b**) and glutamine synthetase (**Figure 4c**) did not differ significantly across treatments. However, the activities of nitrate reductase (**Figure 4d**) in the BF and CF treatments were significantly the highest than those in the control, with increases of 28.4% and 35.5%, respectively.

### 3.7 Investigation of tobacco diseases

The results of the disease investigation are summarized in **Table 5**. Compared to the control, the MR (morbidity rate) and DI (disease index) of bacterial wilt and black shank were significantly reduced in treatments with organic amendments. The DI for the control group were 11.2 (bacterial wilt) and 22.3 (black shank). Specifically, the RFC (relative field efficacies) of BF, CF, PF, and MF against bacterial wilt were 66.7%, 56.0%, 44.0%, and 61.9%, respectively; and against black shank were 66.0%, 52.6%, 42.3%, and 55.7%, respectively.

**Table 5:** Disease investigation of bacterial wilt and black shank.

Treatment		Bacterial wilt			Black shank	
	MR (%)	DI (%)	RFC (%)	MR (%)	DI (%)	RFC (%)
Control	54.5	8.4		9.7	1.1	
BF	25.2	2.8	66.7	3.3	0.4	66.0
CF	29.6	3.7	56.0	4.6	0.5	52.6
PF	33.3	4.7	44.0	5.6	0.6	42.3
MF	26.5	3.2	61.9	4.3	0.5	55.7

BF, biological organic fertilizer; CF, carbon-polymerized organic water-soluble fertilizer; PF, potassium fulvate from mineral sources; MF, microbial fertilizer; MR, Morbidity rate; DI, Disease index; RFC, Relative field efficiency.

### 3.8 The process of soil amelioration by organic fertilizer and its responses and causes for the occurrence of diseases

**Figure 5:**
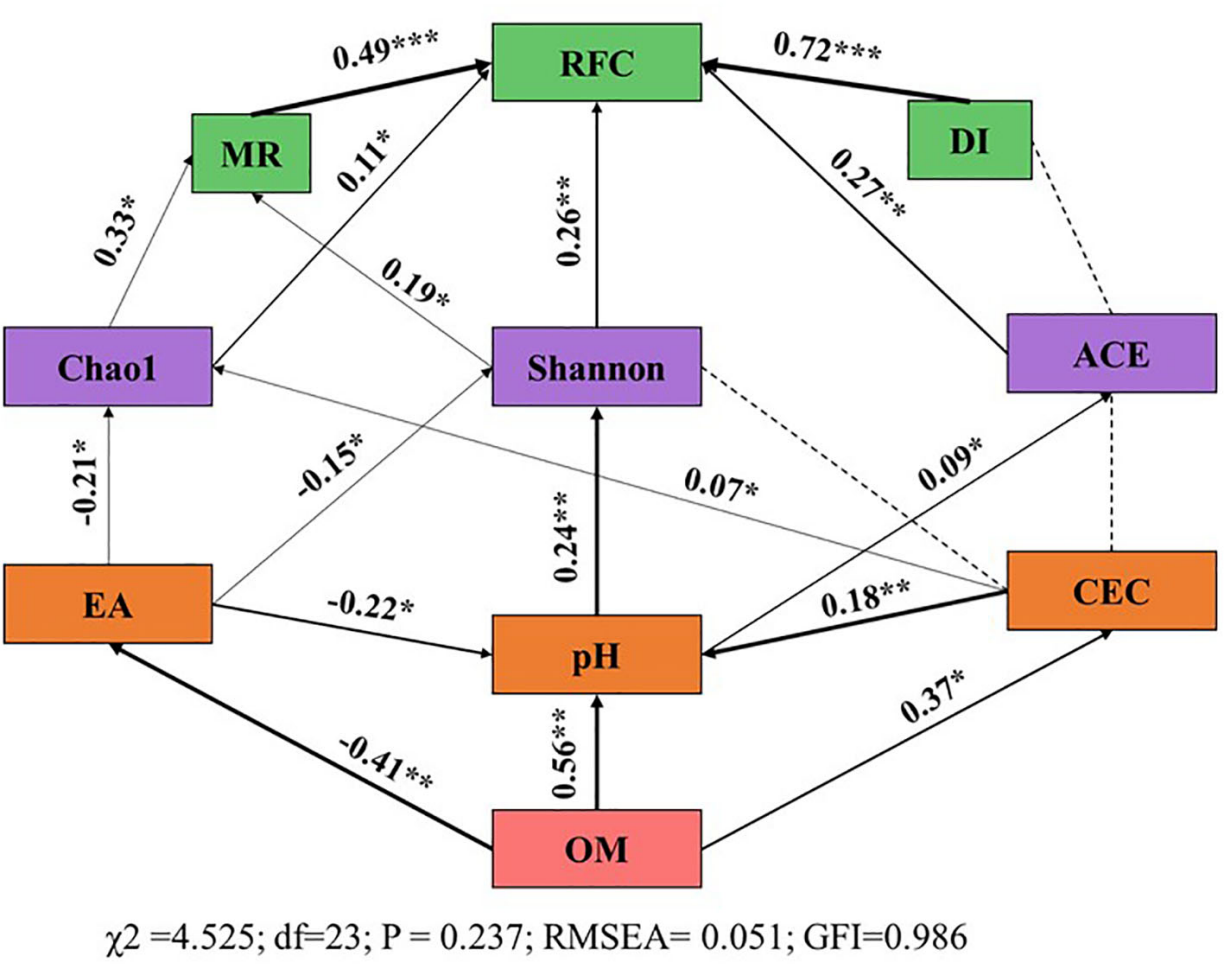
Structural Equation Model (SEM) Analysis of the Influencing Factors of Tobacco Disease Indices. The values associated with the solid arrows represent standardized path coefficients (SPCs), and asterisks indicate the levels of statistical significance: *p<0.05; **p<0.01. BF, biological organic fertilizer; CF, carbon-polymerized organic water-soluble fertilizer; PF, potassium fulvate from mineral sources; MF, microbial fertilizer; OM, Organic matter; EA, Exchangeable acid; CEC, Cation exchange capacity; MR, Morbidity rate; DI, Disease index; RFC, Relative field efficiency.

To elucidate the factors influencing tobacco diseases, a Structural Equation Model (SEM) was constructed to analyze the integrated effects of soil physicochemical properties and microbial diversity on disease occurrence (**Figure 5**). The SEM fitting results were as follows: χ2 = 4.525, df = 23, P = 0.237, RMSEA = 0.051, GFI = 0.986, indicating that the model fit was satisfactory and adequately represented the relationships between independent and dependent variables. The significant increase in soil organic carbon directly reduced acid-producing ions. Soil organic carbon, EA, and EB had direct effects on pH (SPC = 0.56, p < 0.01; SPC = -0.22, P < 0.05; SPC = -0.18, p < 0.01). pH indirectly influenced RFC by directly affecting Shannon diversity (SPC = 0.24, p < 0.01) and ACE indices (SPC = 0.09, P < 0.05), with subsequent indirect effects on RFC (SPC = 0.26, p < 0.01; SPC = 0.27, P < 0.01). EA directly affected Chao richness (SPC = 0.24, p < 0.01) and Shannon index (SPC = 0.09, P < 0.05), and indirectly influenced MR (SPC = 0.33, p < 0.05; SPC = 0.19, P < 0.05). These findings suggest that increasing soil organic matter can mitigate soil acidification (by reducing exchangeable acidity, enhancing cation exchange capacity, and raising pH), promote soil microbial diversity, and consequently reduce disease incidence.

## 4 Discussions

This study investigated the comprehensive effects of bio-organic fertilizer, carbon polymerized organic water-soluble fertilizer, potassium fulvate from mineral sources, and bio-bacterial fertilizer on soil improvement, nutrient enhancement, pH regulation, microbial diversity promotion, microbial community structure optimization, disease incidence reduction, and crop growth promotion. The experimental results demonstrated that the combined application of these four fertilizers had a significant positive impact on soil quality and crop growth.

Soil acidity is classified into active acidity and potential acidity. The pH value represents the active acidity of the soil, while exchangeable hydrogen and aluminum ions indicate the potential acidity. This study found that the application of organic amendments effectively alleviated soil acidification, consistent with the findings of Rukshana et al (Rukshana et al., 2011). This effect may be attributed to the increased chelation of aluminum ions by organic matter released into the soil (Xia et al., 2020), or the promotion of aluminum hydrolysis and subsequent precipitation as aluminum hydroxides (Shen et al., 2020), thereby reducing the content of exchangeable aluminum. Additionally, the decarboxylation process during organic fertilizer decomposition can consume hydrogen ions produced during nitrogen fertilizer nitrification (Rukshana et al., 2011), increase the content of soil organic acid salts, and raise the pH.

There is a strong relationship between soil biota, soil fertility, and plant health (Altieri and Nicholls, 2003). Soil biota play a crucial role in enhancing land productivity and soil fertility through biological processes, which is considered a key strategy for achieving agricultural sustainability (Giller et al., 2005). Soil microorganisms are the most active decomposers in the soil ecosystem, playing a vital role in nutrient cycling and serving as sensitive indicators of changes in climate and soil environmental conditions (Tsiknia et al., 2014). Bacillus-like bacteria, widely used as plant growth-promoting rhizobacteria (PGPR), enhance plant growth and stress resistance (Canwei et al., 2020). Previous studies have shown that Bacillus-like bacteria protect plants by inducing resistance mechanisms and secreting antibacterial substances, thereby promoting plant growth (Grady et al., 2016). This study found that organic amendments containing active microorganisms (BF, MF) had a greater impact on soil microbial community structure and diversity compared to non-biological active organic amendments (CF, PF), consistent with Mawarda et al (Mawarda et al., 2020). This may be due to the large number of exogenous microorganisms carried by organic amendments, which can rapidly colonize the soil and form dominant bacterial communities. Additionally, the organic matter in these fertilizers provides more carbon sources, promoting microbial growth and reproduction.

Soil and rhizosphere organisms are considered biological indicators of soil quality because they are sensitive to minor changes in abiotic stress (Mendes et al., 2013) and influence plant structure, composition, and productivity (Schnitzer et al., 2011). In this experiment, organic fertilizer treatments significantly increased root length, surface area, and volume of crops, and enhanced the activity of enzymes related to carbon and nitrogen metabolism in leaves. This indicates that organic amendments provide a favorable microenvironment for crop root development, indirectly promoting leaf physiological metabolism. The application of organic amendments significantly reduced the incidence and severity of crop diseases and decreased the relative abundance of pathogenic bacteria in the soil. The abundance of *Ralstonia* in the MF treatment showed a significant decrease to 2.5% compared with control (10.5%). This reduction might be associated with the antagonistic activity of *Bacillus subtilis*. Among these, bio-organic fertilizers and microbial fertilizers showed the best effects. This may be due to rhizosphere microorganisms promoting plant growth and protecting plants from pathogens through mechanisms such as antagonism (Mela et al., 2011; Raaijmakers and Mazzola, 2012), nutrient competition (Alabouvette et al., 2006), or inducing systemic resistance by activating plant defense mechanisms (Schenk et al., 2012), serving as the frontline defense against soil-borne pathogens (Cook et al., 1995). The genus Fusarium comprises numerous phytopathogenic species. However, our experimental results demonstrated that the observed population increase in Fusarium spp. did not correlate with a statistically significant elevation in disease incidence. This phenomenon may be attributed to two potential mechanisms: the proliferating Fusarium strains likely represent non-pathogenic variants that occupy ecological niches through competitive exclusion (Mela et al., 2011; Raaijmakers and Mazzola, 2012), or these fungal populations may exert suppressive effects on pathogenic bacterial communities through antimicrobial compound production or resource competition (Alabouvette et al., 2006).

## 5 Conclusion

In tobacco fields affected by continuous cropping obstacles, long-term organic amendments application significantly enhances soil organic matter content, activate soil phosphorus and potassium elements, and improve overall soil fertility. It can increase soil pH, thereby balancing soil acidity and alkalinity. Organic amendments can also augment soil microbial diversity, inhibit the proliferation of pathogenic bacteria, and reduce disease incidence. Additionally, they promote the growth and development of tobacco roots and enhance the activity of carbon and nitrogen metabolism enzymes in leaves. The use of organic amendments creates a favorable microenvironment around tobacco roots, providing a scientific basis and practical model for mitigating continuous cropping obstacles. Taking into account soil improvement, microbial diversity, and disease prevention and control effects, bio-organic fertilizer (BF) demonstrates superior performance.

## Data Availability

The original contributions presented in the study are included in the article/supplementary material. Further inquiries can be directed to the corresponding author.
